# The influence of social networks on self-management support: a metasynthesis

**DOI:** 10.1186/1471-2458-14-719

**Published:** 2014-07-15

**Authors:** Ivaylo Vassilev, Anne Rogers, Anne Kennedy, Jan Koetsenruijter

**Affiliations:** 1 NIHR CLAHRC Wessex, Faculty of Health Sciences, University of Southampton, Southampton SO17 1BJ, UK; 2 Scientific Institute for Quality of Healthcare (IQ healthcare), UMC St Radboud, Nijmegen, Netherlands

**Keywords:** Social network, Self-management, Chronic illness, Network mechanisms, Metasynthesis

## Abstract

**Background:**

There is increasing recognition that chronic illness management (CIM) is not just an individual but a collective process where social networks can potentially make a considerable contribution to improving health outcomes for people with chronic illness. However, the mechanisms (processes, activities) taking place within social networks are insufficiently understood. The aim of this review was to focus on identifying the mechanisms linking social networks with CIM. Here we consider network mechanisms as located within a broader social context that shapes practices, behaviours, and the multiplicity of functions and roles that network members fulfil.

**Methods:**

A systematic search of qualitative studies was undertaken on Medline, Embase, and Web for papers published between 1^st^ January 2002 and 1^st^ December 2013. Eligible for inclusion were studies dealing with diabetes, and with conditions or health behaviours relevant for diabetes management; and studies exploring the relationship between social networks, self-management, and deprivation. 25 papers met the inclusion criteria. A qualitative metasynthesis was undertaken and the review followed a line of argument synthesis.

**Results:**

The main themes identified were: 1) sharing knowledge and experiences in a personal community; 2) accessing and mediation of resources; 3) self-management support requires awareness of and ability to deal with network relationships. These translated into line of argument synthesis in which three network mechanisms were identified. These were *network navigation* (identifying and connecting with relevant existing resources in a network), *negotiation within networks* (re-shaping relationships, roles, expectations, means of engagement and communication between network members), and *collective efficacy* (developing a shared perception and capacity to successfully perform behaviour through shared effort, beliefs, influence, perseverance, and objectives). These network mechanisms bring to the fore the close interdependence between social and psychological processes in CIM, and the intertwining of practical and moral dilemmas in identifying, offering, accepting, and rejecting support.

**Conclusions:**

CIM policy and interventions could be extended towards: raising awareness about the structure and organisation of personal communities; building individual and network capacity for navigating and negotiating relationships and CIM environments; maximising the possibilities for social engagement as a way of increasing the effectiveness of individual and network efforts for CIM.

## Background

Whilst approaches to long-term condition self-management support tend to emphasise changing individual behaviour and improving self-efficacy there is also increasing recognition that self-management (SM) is a collective process, undertaken within social networks and personal communities that requires the mobilisation social resources [1-3]. The literature on the experience of chronic illness consistently points to how people may withdraw from broader social activities and commitments in order to boost or maintain the viability of key domestic relationships. This necessitates shifts overtime in the manner in which people interact with others, leads to changes in contexts, and to renegotiating roles and identities in relations with significant others [4-6]. Other people’s personal experiences have also been shown to help in a number of ways with decisions about chronic illness management [7]. There is evidence too that health behaviours and lifestyle change spread through networks [8,9] and that social networks contribute to long term condition management through the actions, practical, and emotional activities and support work that members of peoples’ personal networks undertake [10,11]. Extending SM to incorporate social network involvement holds out considerable promise for improving outcomes for people with long-term conditions (LTCs). For example there are some suggestions that large, dispersed networks provide access to wider resources [12] and thus potentially act in a positive way for health outcomes through providing access to information [13-15]. Smaller, closed networks may bring benefits through higher frequency interactions and a strong sense of interpersonal obligation. However, evidence for the relationship between social networks and SM remains underspecified as do the practices, mechanisms and resources through which social networks may work in providing support [3,10,16].

The aim of this review was to focus on identifying the mechanisms linking social networks with chronic illness management (CIM). Mechanisms here are understood as the processes and activities taking place within social networks that shape the multiplicity of functions and roles related to CIM that network members fulfil. Here we consider the internal social network mechanisms as located within the broader context of individual and collective chronic illness related practices and behaviours, and with a view to informing the development of policy and interventions.

In this review we included studies dealing with type 2 diabetes SM and/or related health behaviours, risks or associated conditions (multi- morbidity). Type 2 diabetes is an exemplar chronic condition of high incidence and growing prevalence, often co-existing with other multi-morbidities necessitating the adopting and continuation of SM practices. Type 2 diabetes SM is recognised as involving personal behavioural input and support from others (which differs in some respect from type 1 diabetes [17]). Thus, diabetes SMS constitutes a critical case in terms of what might be relevant with to other long term conditions.

## Methods

We used meta-synthesis in order to identify concepts and mechanisms linking social networks and SMS as a technique for the systematic interpretation and re-interpretation of qualitative studies [18,19]. Meta-synthesis is an inductive process through which empirical descriptions and conceptual elaborations across studies are examined permitting novel insights and understandings to emerge from a process of the re-conceptulaisation of themes on three levels. First order constructs constitute the direct feedback of respondents based on their own experiences and interpretations. Second order constructs are interpretations by the authors of the original studies. Third order constructs constitute the final interpretive stage of the synthesis, which is a process of identifying the constructs that best summarise and illuminate the relationship between the research question and the second order constructs. As a method of qualitative synthesis meta-synthesis allows for a deep understanding of the phenomenon under investigation by exploring how it operates within a variety of contexts and in relation to a range of perceptions and influences.

### Search strategy

**Figure 1:**
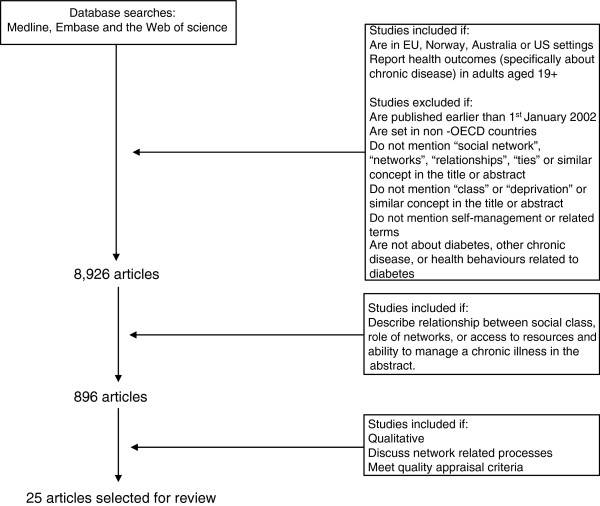
Literature search strategy.

Papers for review were identified from searches in Medline, Embase and the Web of science in order to capture a wide range of studies using four key concepts: social networks, chronic illness, self-management, and deprivation (e.g. social class, inequalities). To achieve cultural and contextual consistency across studies we included studies if they reported health outcomes, practices or behaviours, if the respondents were over 19 years old, if they described the relationship between social networks and the ability to manage chronic illness, if they were conducted in EU, Norway, Australia or US^a^. Due to the large number of papers on these topics and the existence of reviews on the earlier literature we included papers that were published between 1^st^ January 2002 and 1^st^ December 2013. The set of search terms that we used are widely used metaphors and were therefore likely to appear in the main text of studies that were not relevant for this review. We excluded papers that did not mention “social network”, “networks”, “relationships”, “ties” or similar concept in the title or abstract; if the studies were not about diabetes, other chronic disease, or health behaviours related to diabetes; if they were not about self-management or ability to manage disease. For the purposes of this study social networks were understood as personal communities - the set of active and significant ties which are most important to people, with chronic illness in their everyday lives. This included family members, friends, neighbours, colleagues, acquaintances, hobby and other group memberships. Studies about the role of health professionals and user-provider relationships were excluded. 869 papers were reviewed by AK, IV, AR, JK at abstract level (see Figure 1).

All selected papers were discussed by the team with a view of the objectives of the review to illuminate network mechanisms and the content of interactions between social network members (SNMs), and the quality of the research. In assessing the quality of the research we used a quality assessment tool developed by the British Sociological Association [20], which ranks papers as being of high, medium or low quality. Only high quality papers were included for review based on 15 dimensions for quality appraisal summarised as:

• appropriateness of research design to research question;

• relationship of aims and methods to subject and methodological literature;

• systematic, well-considered and documented data collection procedures;

• adequacy of presentation of primary data and its relationship to analysis;

• appropriateness and rigour in analysis.

The results summarised and informed the final selection of articles for inclusion. 25 qualitative papers were chosen for review (see Table 1 below). 14 of the included studies were from US and 12 focused on ethnic minority groups.17 of the papers discussed a broad set of practices, interaction s and behaviour related to type 2 diabetes management, and 8 were focused on lifestyle and disclosure. The studies defined networks in different ways: as family members, 4, relationships with partners, 2, relationships with children, 2, belonging to groups, 2, personal communities, 15.

**Table 1:** Characteristics of papers included in the review

**Study**	**Country**	**Method**	**Sample**	**SM focus**	**Network**					**Study details**
					**Partner**	**Children**	**Family**	**Group**	**Personal community**	
Miller and Davis (2005) [21]	US	Focus groups; thematic analysis	Adults 21-65 with type 2 diabetes; White Americans	General					*	To examine the social support received by people with diabetes and its role in managing diabetes.
			High level of education							
Sparud-Lundin et al. (2010) [22]	Sweden	Individual interviews, constant comparative analysis	13 young adults, and 13 parents, internet communication between young people on diabetes website also included in analysis	General					*	To explore the meaning of interactions and support from parents and other significant others for young adults with type 1 diabetes.
White et al. (2007) [23]	Ireland	Focus groups, thematic content analysis	4 patients with good HbA1C control) and 4 family members, median age 75; and 5 patients with poor HbA1C control) and 6 family members, median age 67; Older adults, type 2 diabetes	General			*			To explore the beliefs, attitudes and perceptions of adults with type 2 diabetes and their family members.
Beverly et al (2008) [24]	US	Focus groups; thematic analysis	30 couples (person with diabetes and spouse); Middle-aged and older adults	Dietary changes	*					To determine how aspects of the spousal relationship translate into behaviour changes, especially adherence to a healthy diet.
Stone et al. (2005) [25]	UK	Semi-structured interviews; framework analysis	20 respondents with diabetes; South Asians	General					*	To explore the experience and attitudes of primary care patients with diabetes living in a UK community with a high proportion of South Asian patients of Indian origin, with particular reference to patient empowerment.
			White British							
Gorawara-Bhat et al. (2008) [26]	US	Open ended semi-structured interviews; thematic analysis	28 people with diabetes (66-87 years); African A	General					*	To explore the role of social comparison with peers/family members in the self-management practices of older diabetes patients.
			Women							
			(predominantly)							
Chesla and Chun (2005) [27]	US	Group interviews, narrative and thematic analysis	20 participants (person with diabetes and spouses) representing 16 families; Chinese Americans	General			*			To describe family responses to type 2 diabetes in Chinese Americans as reported by people with diabetes and spouses.
Beverly and Wray (2010) [28]	US	Focus groups; thematic analysis	30 couples (persons with diabetes and spouses); Middle-aged and older adults	Exercise adherence	*					To illuminate the potentially key role of collective efficacy in exercise adherence in order to develop and test interventions that provides more effective support for adults with diabetes.
Laroche et al. (2009) [29]	US	Semi-structured interviews; thematic analysis	24 adults (19 parents and 5 grandparents) with diabetes and child (10-17 years), and 24 children (12 male and 12 female); African A	General		*				To examine the role of children in their parents’ diabetes self-management, diet and exercise.
			Latinos							
			(inner city)							
Gallant et al. (2007) [30]	US	Focus groups; thematic analysis	13 focus groups with 84 (65 years or older) with arthritis, diabetes, and/or heart disease; African A	General					*	To contribute to knowledge about older adults with chronic illness by identifying positive and negative influences of family and friends on self-management.
			White A							
Carter-Edwards et al. (2004) [31]	US	Focus groups; thematic analysis	3 focus groups, 12 African American women with diabetes (average age 49.3); African A	General			*			To evaluate the relationship between perceived social support among African American women with type 2 diabetes and self-management.
			Women							
Ruston et al. (2013) [32]	UK	Semi-structured interviews; constant comparative method	43 respondents (23 female and 20 male); Work environment, employees	General				*		To explore the perceptions and experiences of employees with diabetes.
Jones et al. (2008) [33]	US	Focus groups; thematic analysis	21 people with diabetes 6 and family members/friends (27-85 years); African Americans	General					*	To examine the impact of family and friends on the management of persons with diabetes.
Sarkadi and Rosenqvist (2002) [34]	Sweden	Individual interviews and focus groups, thematic analysis	5 interviews and 5 focus groups with 38 women, 44-80; Women	General					*	To systematically investigate the conflicting demands of social network involvement with illness management on women’s type 2 diabetes.
Essue et al. (2010) [35]	Australia	Semi-structured interviews; qualitative content analysis	14 carers (45-85 years) of people with chronic heart failure, COPD, and diabetes	General			*			To describe the family careers’ contribution to the self-management partnership and To identify policy and practice implications that are relevant to improving the support available for informal care in Australia.
Laroche et al. (2008) [36]	US	Semi-structured interviews; thematic analysis	29 interviews (14 adult-child pairs and one child); African A	Diet		*				To explore how adults with diabetes attempting to change their own diets approached providing food for their children and how their children reacted to dietary changes in the household.
			Latinos							
			(inner city)							
Kohinor et al. (2011) [37]	Netherlands	Semi-structured interviews; grounded theory	32 diabetes patients (36-70 years); Surinamese	Disclosure					*	To explore why diabetes patients from ethnic minority populations either share or do not share their condition with people in their wider social network.
Kokanovic and Manderson (2006) [38]	Australia	In-depth interviews; thematic analysis	16 immigrant women with type 2 diabetes; Immigrant women	General					*	To elucidate the social meanings and interpretations that immigrant women attach to the diagnosis of type 2 diabetes, and the social support and professional advice they receive following this diagnosis.
			Greek, Chinese, Tongan, Indian							
Atkinson et al. (2009) [39]	US	Focus groups, grounded theory	4 focus groups in churches in south-eastern US, 3 with church leaders and one with programme participants; African Americans	Healthy lifestyle; diabetes prevention				*		To explore church members’ perspectives of implementation of church-based diabetes prevention programme with African American churches.
			Church members							
Chlebowy et al. (2010) [40]	US	Focus groups; content analysis, thematic analysis	38 adults (27 women, 11 men), 44-87 years, 7 focus groups; African Americans	General					*	To identify facilitators and barriers to self-management of type 2 diabetes mellitus among urban African American adults.
Jepson et al. (2012) [41]	UK	In-depth interviews and focus groups; thematic analysis using both inductive and deductive coding	59 purposefully selected Bangladeshi, Indian, and Pakistani; and 10 key informants; South Asians	Physical activity					*	To explore the motivating and facilitating factors likely to increase physical activity for South Asian adults and their families.
Pistulka et al. (2012) [42]	US	Qualitative interviews; constant comparative method	12 participants (8 women and 4 men), 40-65 years, 12 face to face interviews and 6 follow up follow up interviews; Korean American Immigrants	General					*	To examine the illness experience of Korean American immigrants with diabetes and hypertension.
Shaw et al. (2013) [43]	US	Focus groups and interviews; thematic analysis	3 focus groups and 5 interviews with 13 adults with type 2 diabetes; American Indian/Alaska Native Adults	Diabetes					*	To explore perceived psychosocial needs and barriers to management of diabetes among AI/AN adults with type 2 diabetes.
Thompson et al. (2013) [44]	Australia	Ethnographic and participatory action research; unstructured and semi-structured interviews; thematic analysis	23 purposefully selected community members over 16 years; Indigenous people	Physical activity					*	To explore and describe local perspectives, experiences and meanings of physical activity in two remote indigenous communities.
Ward et al. (2011) [45]	Australia	Semi-structured interviews; content thematic analysis	Participants with diabetes (17), COPD (3) and/or CHF (11), and family carers (3); Aboriginal and Torres Strait Islander people	General					*	To explore the lived experiences and to uncover the ways in which Aboriginal and Torres Strait Islander people with chronic illness experience informal unsolicited support from peers and family members.

*Main focus of network discussion in the paper.

The review follows a line of argument synthesis where concepts across studies are translated into one other in order to map and interpret them [18,19]. Extraction forms were used for analysing and systematising the data. This included background of the studies, quotes from respondents, interpretations and analysis by authors, references to social networks, key findings, and interpretations and comments by reviewers. The review process included an initial stage where three papers were analysed by all authors AR, AK, IV, JK. The remaining papers were then split between the authors and analysed individually (and by at least two people). All authors subsequently discussed the findings. Different visualisations on whiteboards and on paper were used in order to experiment with different groupings and links between concepts. This process went through a number of iterations before the final conceptualisation of second order constructs was agreed, and the structure and organising principles of the third order synthesis finalised.

We kept a record of and revisited decisions taken earlier and discussed conceptualisation and interpretations of the data at project meetings with colleagues involved with the EU-WISE project of which this metasynthesis was a part.

## Results

### Network involvement in illness management: second order synthesis of concepts

Three themes were identified and illuminated how engagement with network members shaped people’s experiences, expectations, and processes of managing a long term condition.

#### ***Sharing knowledge and experiences in a personal community***

Sharing knowledge and experiences within a personal community can provide people with a sense of not being alone and offers a valued opportunity to exchange gain and reinforce existing knowledge relevant to a condition [21,22,42,43,45]. The process of sharing also feeds into people’s internal capacity to cope with stress and, though not always explicitly acknowledged, can act to motivate lifestyle changes or involvement by adding new activities with which to self-manage [21-23]. The motivation to undertake activities such as regular exercise, program attendance, and dietary change is linked to a sense of shared accountability for doing things together with people who are both familiar and trusted [24,41,44]. In some circumstances, this is reversed and the sharing experiences can provoke anxiety which can also become a shared network phenomenon if for example they get a sense that other network members are failing where they fail in understanding available information [23,25].

People with LTCs make changes and adaptations by observing what others do, social comparison, and modelling on others with similar conditions [21,24,26,27,41]. These can have both positive and negative impact [26,45]. When poor outcomes are observed in other network members this can lead to the seeking of support from elsewhere in order to prevent similar outcomes [21,24]. However, it is also the case that comparison with non-ill network members can impact negatively on one’s sense of well-being and their efforts to improve their health [26]. The presence of family or network histories and experience of diabetes enhances awareness of diabetes making it more likely that there will be an accumulated stock of relevant illness knowledge within the group. However, the latter in some circumstances may lead to resignation about being diagnosed to taking action or being motivated to change [25].

Network members can shape the behaviour of people with LTCs through providing cues to action, indirect coaching, or using covert ways to influence behaviour. This might involve a third party mediator to encourage change [27] or reference to examples and stories of health relevant practices in communicating and discussions with the individuals. A more direct means of influence is through providing advice on how to improve outcomes [21,24,28,39,40]. The nature of the relationship is relevant in determining influence. Paradoxically, the influence that strong “bonding” ties (of partners and close family), which are intimate emotional, frequent and intense, could have limited impact because their concerns and advice might not be taken seriously by the person with a LTC [28]. Network members’ influence is seemingly limited whenever formal medical knowledge associated with professionals is perceived as superior to experiential and network based knowledge [25]. Given that contact time with professionals tends to be short and infrequent this limits the possibilities to integrate or link professional advice with pre-existing illness network knowledge, experience and capacity.

Network influences can be both positive and negative [29,43]. However, overall in the literature there tends to be more positive network influences noted than negative ones, and more negative influences from family members than from friends [30]. This might be related to the inherently more problematic potential for making changes in one’s family than non-family networks. Network structure tends to evolve, with negative influences in particular, being dropped over time, so re-shaping one’s network is far more difficult to do with family members than it is with friends, neighbours, colleagues, or other ‘weak ties’ [30,39].

Collective efforts make it easier for people to make changes [40,41,44], and influences run in both directions in networks. Thus, network members sometimes adopt changes themselves not only deliberately and strategically, but also unreflexively through incremental change in their own routines. However, there are limitations to the possibility of collective effort and change as network members are obviously limited by their physical abilities or lack of knowledge [28,29,43]. Access to diverse network members is more likely to have a positive effect as it increases the likelihood that a network member with a similar level of physical capacity, interest, and willingness to make specific changes would be accessible.

#### ***Accessing and mediation of resources***

Network members provide overt forms of support to illness management activities such as monitoring, medication management, checking blood sugar, reminders, shopping and meal preparation, physical activities, health care appointments, decision-making about the illness, psychosocial coping and emotional support [21,25,29,30,40,43-45]. This is dependent on network members having the relevant knowledge and ability to do this competently [22,31]. As the existing knowledge available from network members can be rudimentary and insufficient to address illness management needs [25]. Additionally accepting support from network members can be experienced as more challenging and difficult when this lies in work settings where there maybe concerns about being stigmatised or treated inappropriately [32].

Limited access to formal healthcare resources can lead to higher dependence on personal network members for material help and psychosocial support [33] and the use of network support is potentially burdensome as it is accompanied by expectations and obligations as well as an awareness of the restrictions (such as time and obligation to provide help on an ongoing based) which may be imposed on network members as a result of providing support. In this respect, the extent of network support is sometimes invisible and under-acknowledged by people with LTCs, possibly as a way to reduce stress levels related to perceptions of unfulfilled responsibilities to others [23].

Some papers point to how network members can create obstacles to obtaining resources for illness management due to lack of understanding about the specific regimen associated with the illness, food choices and diet or by creating an environment that creates barriers to the needs of people with LTCs (e.g. the raising of unrealistic expectations requiring physical activity) [23,33,45].

#### ***Self-management support requires awareness of and ability to deal with network relationships***

Living with a chronic condition shapes relations with network members at home, work in social situations and the quality of life of oneself and other network members [24,27,31,33,34,42,44]. The alignment of individual and group objectives and priorities involves balancing the objectives of illness management with other valued social roles, such as being a partner, parent, child, friend, colleague [24,27,45]. It involves managing the concerns, demands, and expectations of network members, around food and medication, and around adapting to existing and new roles that network members perform, including being a home help, lifestyle coach, advocate, technical care manager and health information interpreter [21,24,35]. Negotiations about these roles and functions can take different forms, for example, parents with a LTC might demand lifestyle changes from their children through concern over them developing the condition in the future [36].

Network members relate in a variety of ways to a person’s illness ranging from considering diabetes as being ‘not a real illness’, through accepting the illness, to over-concern and over-control. This can create challenges for the management of relationships within networks where there is blame and stigma concerning personal responsibility and body image [22,23,26,31,34,37,42]. However, it is concern by and for others rather than lack of concern that is forefronted by people with LTCs [29,42,44,45]. Maintaining a sense of autonomy and control over one’s life and a sense of equal and reciprocal relationships is highly valued but often threatened due to diminished capabilities and/or over-concern and vigilance, and heightened perception of illness severity by other people [21,22]. Over-concern can also be a threat in the work environment if the illness is interpreted as a barrier to fulfilling one’s work responsibilities [34].

Managing the responses of other network members is motivated by reciprocal concerns over the well-being of colleagues and not wanting to be a cause of unnecessary worry. Accepting assistance is also a balancing act requiring considerations of the demands on other people’s time, resources, and other roles they might have to fulfil [22,27,29,31]. Increased demands and concerns might also lead to carer self-neglect [35]. Given these factors, relationships with others cannot easily be taken for granted and maintaining them is an active process requiring careful vigilance when managing disclosure to different network members or deciding who to seek help and advice from. Existing network resources are also not necessarily cumulative as accessing one type of support may restrict access to other network members.

Styles of engagement between someone who has type 2 diabetes [4] and their network members range: from avoidance and concealment to openness and direct engagement. For example, people with LTCs might avoid conflict or discomfort by avoiding disclosure [21,27,34,42]. Open and direct engagement with social network members is more likely in the presence of a shared sense of confidence, expectation and social cohesion [21,28,30,38]. Direct engagement opens up possibilities for [22,24] building collective understandings and support as a team effort, which in turn creates a supportive health environment [24,31,33]. For example, this could be in terms of adherence to dietary regimen, joint shopping and consideration of what food is cooked and how [30].

The expression of a broad concern for a person’s well-being and acknowledgement of achievement may encourage beneficial changes to existing practices [21,22,24] whilst over-vigilance on needing to manage an illness could have a negative impact on a persons’ sense of well-being [21,27] and relationships with network members [22]. The possibility of individual change is closely dependent on changes within the environment within which one operates with others. For example, people with LTCs find it easier to make changes when network members eat the same meals and make changes to the routines of their own daily lives adhere to similar decisions sustaining behavioural changes, and through accepting a change in their own roles [21,22,29].

### Illuminating network mechanisms in chronic illness management: third order synthesis

Three concepts emerged from the process of interpretation and further synthesis of the second order constructs which illuminate the mechanisms linking social networks and health relevant outcomes. These are *network navigation, negotiating relationships*, and *collective efficacy*. Additional file 1 shows the relationship between second and the third order concepts. *Network navigation* refers to identifying and connecting with relevant existing resources in a network. It involves, making decisions about when and who to contact, identifying and utilising resources that were previously underused, concealing the selection of some ties over others, and building justifications that successfully preserve existing relations.

Our metasynthesis captures the requirement over and above navigation to *negotiate* and re-negotiate existing relationships, roles, expectations, means of engagement and communication between network members. This involves judgments about which relationships require reshaping, strengthening, abandonment, and new ones developed. The process of *negotiating relationships* within networks requires building justifications of responsibility, and level and type of involvement. *Network navigation* and *negotiating relationships* bring to the fore the need for the fulfilment of expectations of reciprocity, complexities of availability and acceptability of support. It is clear from this review that approaching network members for help is not exclusively based on their knowledge and capacity but is an aspect of the relationship and moral identity work that take place within the network. For example, the desire for independence and autonomy may take precedence over needs for assistance, and may be a reason for not activating support networks even when they are available [16,46].

**Figure 2:**
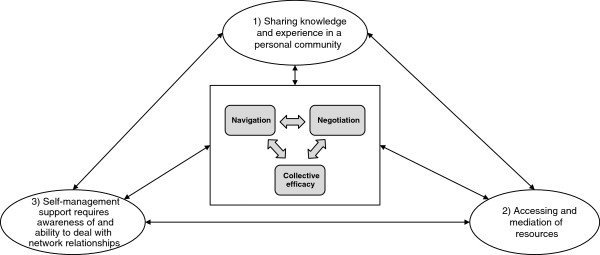
**Summary of 2**^**nd **^**and 3**^**rd **^**order concepts.**

**Figure 3:**
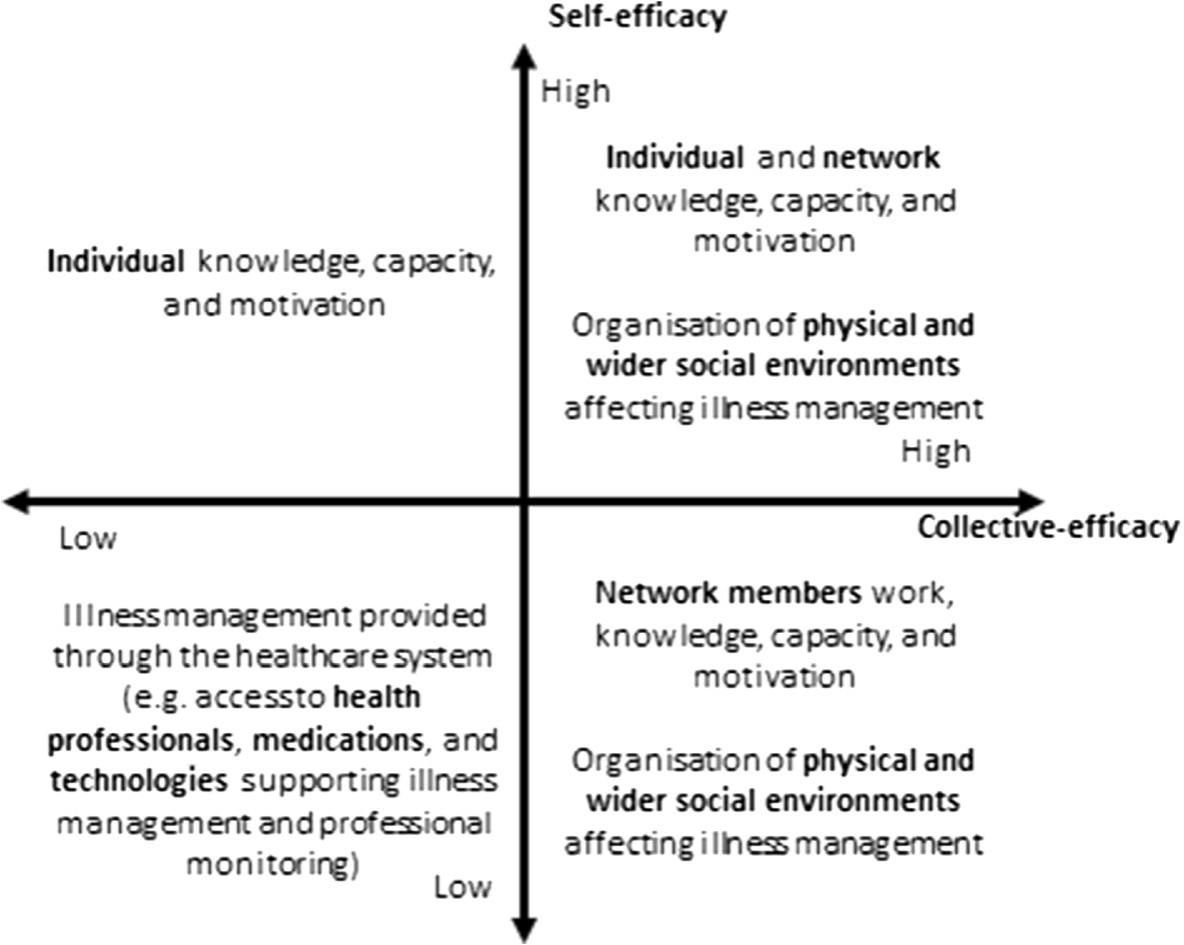
Self-efficacy and collective efficacy.

The involvement of network members in illness management forms an aspect of a collective network process, effort and change placing emphasis on collective agency rather than individual *self-efficacy. Collective efficacy* can be understood here as a shared perception and capacity to successfully perform and behave through shared effort, beliefs, influence, perseverance, and objectives (Figure 2). *Collective efficacy* can be limited to one or two network members, or be spread across an entire personal community and the wider set of groups that individuals belong to (e.g. place of work, locality).Identifying the significance of collective efficacy brings with it a set of continuities and tensions with the current normative and policy emphasis on self-efficacy as a way of improving illness management (Figure 3). Four broad scenarios for illness management can be identified: low self-efficacy/low collective efficacy, high self-efficacy/low collective efficacy, high collective efficacy/low self-efficacy, and high self-efficacy and high collective efficacy.

The four possible scenarios modelled above illustrate that making a choice about illness management policy and interventions involves nuanced political and value choices, and affects differently the interests of stakeholders. For example, interventions focussed on improving motivation and individual knowledge tend to work best for people who are already motivated and knowledgeable, and to be less effective in deprived populations.

## Discussion

Previous research on social networks has been instrumental in implicating the importance of network effects for different health related outcomes including self-management [11,12]. The meta-synthesis undertaken here clarified aspects and mechanisms which are relevant to personal support for the management of a LTC (type 2 diabetes). Our findings indicate that social network involvement with CIM is related to the distribution of illness work that SNMs take over or share the burden of. Network members influence things via a number of means- through sharing knowledge and experience, observing, making comparisons with, and modelling on what network members do. In this respect SN members can be conceptualised as an active extension of the person with a LTC complementing and adding to their efforts and capacities in completing illness management tasks. However, network processes are rarely one-directional. The work that network members do for a person tends to be reciprocated with network influences running in both directions.

In common with other studies [3,47] the involvement of social network members is not unambiguously related to positive influences [8,16]. Engagement with one’s network implies the necessity of carrying out relationship and identity work. Whilst engagement with social networks can lead to change, it can also create obstacles to change and positive as well as a negative impact on people’s health and CIM or highly selective impacts. For example, providing help with practical everyday tasks reduces the amount of work that people with LTCs need to do themselves, thus opening more time and leaving more energy to completing other activities. These could include illness monitoring tasks, medication taking, doing physical activities, and keeping social involvement. However, accepting support may also lead to a sense of losing control of one’s life and autonomy or if network members provide more support than the person wants or needs this may prevent the use of their full physical and mental capacity to develop sustainable illness management strategies. These complexities in network dynamics offer an insight as to why network support cannot simply be reduced to a cumulative process (i.e. more network members more network support) even where a degree of substitutability between network member support might exist [11]. Access to different types of network members offers access to a wider range of information sources and support [13,14], opening possibilities for adaptions to be made in relation to individual identities, concerns preferences [6] and context.

The network mechanisms that we identified are broadly related to individual and network members’ capacity of *network navigation* and *negotiation* and *collective efficacy* created by network members. Our review suggests a janus face of the role of networks which are characterised by contradictions irreconcilable objectives, outcomes, roles, identities, values inherent which can vary across the contexts within which CIM takes place. Nonetheless, network navigation can improve access to relevant knowledge and resources, while allowing people with LTCs to avoid potential conflicts and preserving valued roles and identities. How network mechanisms relate to CIM is shaped by the environments in which they take place which can be enabling or disabling depending on the capacities they offer for carrying out illness management work and supporting behaviours beneficial for people’s health. In this respect illness management environments are organised around a variety of logics: evolution of domestic relationships in the home and the needs of the household, the objectives of employers, the need of private sector companies to make profit. These are potentially open to external intervention and can be orientated towards making illness management and people’s health needs a higher priority [48,49].

## Conclusions

This qualitative meta-synthesis examined the mechanisms linking social networks and illness management which has brought into view the way in which illness management (more usually construed as an individual behavioural phenomenon) is a collective process and takes place in a context of multiple objectives and values that are interrelated. We identified three key social network mechanisms which have utility in considering the nature of future chronic illness management strategies. Network processes of importance might include more active navigation of some network involvement and the changing priorities within specific environments, including the avoidance of places and relationships that can trigger undesirable situations and enhancing those that have more positive influences. Drawing on the notions of collective efficacy and enabling environments we identified set of continuities and tensions within the currently dominant normative and policy emphasis on self-efficacy as a way of improving illness management (see Figure 3 above).

Our findings are likely to have implications for policy development as they indicate that the current focus on self-efficacy could be extended towards raising awareness about the structure and organisation of personal communities, building individual and network capacity for navigating and negotiating relationships and SM environments. In this respect interventions could be more productively designed to maximise the possibilities for social engagement, particularly through extending people’s access to weak ties and the building of enabling environments that have relevance for illness management.

### Study limitations and future research

This metasynthesis only included qualitative studies. This approach has advantages as qualitative studies offer access to understanding the underlying mechanisms through which social networks operate and fills a gap left by quantitative systematic reviews. The limitations of this review are that the concluding picture presented of network involvement (of the three mechanisms) are limited to a set of propositions which require testing out in empirical studies. Additionally, whilst this metasynthesis was primarily focused on understanding the mechanisms through which social networks are understood as relationships outside formal healthcare operate this necessarily excludes the impact of professionals and the structure and extent of network involvement in illness management which is shaped by the organisation and funding of formal healthcare provision and the ethos of professional-user relations. Future research would need to illuminate illness management at the interface of personal communities, healthcare system support, broader social and physical environment, and individual self-management.

### Ethics statement

The paper is a metasynthesis of published studies all of which had ethical approval.

## Endnote

^a^The latter was because this review was a part of an international project including six European partner countries and in order to include the countries where most of the research on SM has been carried out.

## Competing interests

The authors declare that they have no competing interests.

## Author’s contributions

AR designed the study, IV and JK carried out the literature searches, AR, AK, IV, JK selected the papers for review and reviewed the final papers, IV wrote the first draft of the paper, AR, AK, JK, IV revised and finalised the paper. All authors read and approved the final manuscript.

## Pre-publication history

The pre-publication history for this paper can be accessed here:

http://www.biomedcentral.com/1471-2458/14/719/prepub

## Supplementary Material

Additional file 1 **Examples of 2**^**nd**^** and 3**^**rd**^** order themes.** Click here for file
