# Supplementary material for: The influence of social networks on self-management support: a metasynthesis
Source: BMC Public Health. 2014 Jul 15;14:719. doi: 10.1186/1471-2458-14-719 (PMC4223639; doi:10.1186/1471-2458-14-719)
Supplement: Additional file 1 — Examples of 2 nd and 3 rd order themes. [file 1471-2458-14-719-S1.doc]

**Additional file 1: Table S2.** Examples of 2^nd^ and 3^rd^ order themes

| Example concepts from second order extraction | Quotes from papers | Translated themes (second order synthesis) | Line of argument (third order synthesis) |
| --- | --- | --- | --- |
| 1) Sharing knowledge and experiences in a personal community  Personal awareness of diabetes risk seen as an initial impetus to involvement in the program and individuals’ willingness to make lifestyle changes. Awareness promoted through testimonials from others, endorsement from church leadership, consideration of positive screening results, and educational materials [24]  Contradictory interchange of diabetes experiences –confirms shared reality of diabetes experience which is valued when sought, but young people do not like forced group encounters [22]  Felt ‘understood’ by other people with diabetes [38]  Respondents wanted family members to change their behaviour as a means of supporting them e.g. eating habits which make it easier for them to comply [40]  An external downward comparison, which is empowering and sustains good eating control by the patient… caution needed not to use comparisons to the extent that they lead to stress and making patients feeling they are outperformed…Barriers to health outcomes are when comparisons are made to normal (i.e. non-diabetic) network members and behaviours are geared towards maintaining sense of well-being [26]  Poor outcomes in network members lead to seeking of informational support to prevent similar outcomes [21]  Indirect coaching of behaviour in the person with diabetes hidden or oblique ways of influencing behaviour. Using personal modelling as form of persuasion, using indirect communication through significant others, highlighting roles and responsibilities to stay healthy [27]  The diabetes prevention group provided a network of newly formed relationships that provided opportunities to exercise together, share nutritional information, and provided a sense of shared accountability with respect to regular exercise, program attendance, and dietary change [24]  Support from family, peers, and health care providers positively influenced adherence behaviours by providing cues to action, direct assistance, reinforcement, and knowledge [40]  Health professionals provide appraisal for self-management practices and informational support on how to improve outcomes [21]  Personal awareness of diabetes risk seen as an initial impetus to involvement in the program and individuals’ willingness to make lifestyle changes. Awareness promoted through testimonials from others, endorsement from church leadership, consideration of positive screening results, and educational materials [39] | “I’m very fortunate there’s a co-worker that is type 2 diabetic and we’re talking to each other all the time about hey, how’s your blood sugar doing? How’s the last doctor visit?”  “One of the guys at work is going through the same thing…it’s kind of like a little support group that we encourage each other to keep going” [21]  “My diabetes is under such excellent control because I know my friends; they eat such horrible things” [26]  “…my aunt never drank,…never smoked,…never kept hours…ate well…and it’s paying off for her…she’s in excellent health for her age…and I mostly want to be independent like her…” [26]  Black male: “I have a bad example in my household. My 98-year-old mother in-law eats everything in sight and is not happy unless she’s had three meals a day so we have three meals a day at our house, big meals, and I can’t stand to see food around and not eat it. That influences me negatively.” [30]  ‘Even close family or close friends do not understand what has happened to me in so short a time. Because they are used to the old me still, you know. I think that only people who experience it (diabetes) can understand’ (Sarika, Indian).  “I have learned about life and I feel different because when I came here [diabetes support group], I talk to people and learn things. I know other people with diabetes. We talk [about] what tablets one [person] takes, what tablets the other takes. It is helpful because we get each other’s opinion”. (Evania, Greek) [38] | Sharing similar knowledge and experiences  Upward and downward comparisons  Modelling on/learning from others  Validation, endorsement and support by others | *Network navigation*  People with LTCs and network members make judgements of when and who to contact and which relationships require strengthening or adopting and which ones need to be abandoned  *Negotiation of relationships*  network members engage in a process of rebalancing relations and negotiating roles and objectives through narratives of responsibility and through negotiating levels and types of involvement  *Collective efficacy*  a group’s shared perception and actual capacity to successfully perform behaviour through a shared effort, beliefs, influence, perseverance, and objectives |
| 2) Accessing and mediation of resources  Providing meals that were appropriate for the care recipients’ dietary needs meant that carers develop an understanding of what was required in the new diet and an ability to provide the diet at the required times [35]  Spouses frequently the key provider of social support, helps to provide constant vigilance [21]  Parent becoming guiding agent, contributing knowledge and competent, trusted advice – reminder of social capital issues and their supporting role shifts [22]  Importance of understanding the management of diabetes as a collaborative team effort, for rural African Americans, who often have limited access to health care resources and count on family and friends for material help and psychosocial support [33]  The need to be persistent in seeking support is hard to maintain [21]  Concerned but unqualified partners – lacking experience implies insufficient competence [22]  Lack of understanding – from family members about type 2 and therefore unable to be of help when they wanted this…general stress of life as multiple non health care givers makes it difficult physically and emotionally to maintain effective diabetes management [31] | ‘I didn’t know everything has salt . . . I mean, it was incredible, I must have spent an hour in every aisle . . .reading the labels for potassium and sodium.’  ‘[I] do all her tablets for her . . . she can’t manage the shopping any more . . . I’ll take her there and carry the stuff, she wouldn’t be able to go down to the shops [unassisted].’ [35]  White male: “My wife takes care of my pills for me.”  Black female: “And she [daughter-in-law] does my medicine. She got these different things to put my medicine in for an entire week . . . because I can’t do it. If I do it, she checks it.”  White male: “My wife keeps asking me, did I take my medicine?”  White male: “When we go out or travel anyplace, the first thing my wife will ask me, ‘Do you have your vampire kit?’ which is my glucose meter, and ‘Do you have your insulin?’ because a couple of times we’ve gone out to dinner and I forgot to take the insulin.” [30] | Network members taking over aspects of illness work  Network support as a safety net  Work involved in getting and sustaining support  Limits to network support |
| 3) Self-management support requires awareness of and ability to deal with network relationships  Accommodation in family encompassed via a balancing act between disease management and quality of life and between attention given to ill person and other familial demands social roles and obligations…. Maintained ease of social relations with family despite diabetes symptoms and care requirements [27]  Partners can take over parental role, potentially causing conflict and challenging identity as couple [22]  For family networks – women see their health as contingent on the wellbeing of the entire family – her health-related behaviours secondary to this. Women non-compliant with treatment that does not fit day to day way of life preceding diagnosis. This leads to stress and anxiety. Role demands interfere/conflict with self-care regimens [34]  Spousal control over food led five people with diabetes to stash food in the house and caused considerable conflict with the majority of couples [24]  Barriers extended beyond the immediate program participants to include others in their social networks. Initially participants struggled with family and social expectations and resistance to changes in role-related tasks (e.g. food preparation) [24]  Diabetes not always perceived as real illness [34]  Patients had a sense of control over diabetes – relaxed attitude …Family members had heightened perception of severity of diabetes, increasing their concerns [23]  For both patients and family members a perceived lack of information and an inability to understand the information they get – leading to patients stopping asking questions [23]  There is a gender bias and prejudice in the way women and men are dealt with by health professionals – women’s symptoms judged negatively as being psychological this leads them to feeling helpless in medical encounters. Often a gender-role game going on – women need to assert themselves more particularly with female practice nurses [34]  no disclosure to people outside immediate family due to taboo and shame [37]  measuring blood glucose and undertaking disease-related regimes at work troublesome – done secretly (or not at all) and diabetes concealed because of threats to losing job or being viewed as unclean or unsuccessful woman (because of dealing with blood and urine)…Do not want to share information about diabetes with co-workers, elements of diabetes regime are troublesome and concealed [34]  no disclosure to close family not to worry them [27]  Women not wanting to be a burden on loved ones [31]  using indirect communication through significant others, highlighting roles and responsibilities to stay healthy [27]  Conflict in disclosing diabetes at work may compromise disease management [34]  Aspects of concealment and revelation to others. Practices hidden from family members selecting foods discretely [27]  Some young people neglect support, others actively seek positive support and not moralising [22]  Most effective strategy for support was asking for it directly… Constant vigilance of eating happens frequently. May be a negative aspect of social networks and depress wellbeing feelings [21]  Participants needed to develop strategies for obtaining support that led to better control [21]  Managing friends’ involvement – a spectrum – empathy for wellbeing and practical help for emergencies are valued, but some value independence over supportive actions [22]  Women not wanting to be a burden on loved ones [31]  Family members notice changes and all have to adapt to the impact on day to day life [23]  maintenance of personal change was dependent on complementary and supportive changes in the participants’ family and church community support from the church and their immediate friends and families was important in sustaining their healthy behavioural changes [24] | ‘So I think that this conflict can be the result of more, this anxiety you have, for diabetes is actually a devil disease’  ‘Think if you’d got it in the car and crashed. She’s right in what she says but I can’t handle her. She also says ‘you have to eat right away after training, before you get in the car, a banana and juice isn’t enough’.  I think it’s enough with what I eat until I get home and dinner is ready. Yeah, then the fuss has started. Both of us want to be right and refuse to give in. Yeah, Yeah but it’s beginning to settle now. Just needed to talk about it!! Was so damn angry now tonight!’ [22]  ‘It could be a barrier to my leadership. For the beginner Christian, it could be a question that, as a good Christian like me, how come she could be suffering with DM, although she always prays and she looks to have a special relationship with the Lord . . . . I cannot say that this [DM] was given to me by the Lord.’ [42]  ‘Would people come to exercise in a pool?  Alice: Well I think I don’t like (doing this) … It’s different like, other people might think “oh these black people don’t usually do that swimming around and exercising, running or jogging … (Bininj woman, 36-45)’ [44]  ‘…rather that than he doesn’t bother about it, that would feel like he doesn’t care about me as a person…’. [22]  ‘Maybe I was afraid. Once I revealed my problems, I would appear weaker, so maybe I had to keep it under wraps and make it look like everything’s under  control . . . . I didn’t want them to see me ill.’ [42] | Maintaining normality, roles, expectations  Conflicts in roles and relationships  Interdependences between changes within networks  Illness perception, meaning of illness  Encounters with healthcare professional  Self-identity, body image and stigma  Care and concern for others  Avoidance and concealment  Openness and direct engagement  Indirect engagement  Selective engagement  Interdependences between individual and collective change |
